# An assessment of neuronal calcium sensor-1 and response to neoadjuvant chemotherapy in breast cancer patients

**DOI:** 10.1038/s41523-018-0057-7

**Published:** 2018-03-12

**Authors:** Lauren M. Moore, Rachel Wilkinson, Mehmet Altan, Maria Toki, Daniel E. Carvajal-Hausdorf, John McGuire, Barbara E. Ehrlich, David L. Rimm

**Affiliations:** 1 Department of Pathology, Yale School of Medicine, New Haven, CT USA; 2 Department of Pharmacology, Yale School of Medicine, New Haven, CT USA; 3 Department of Thoracic/ Head & Neck Medical Oncology, The University of Texas MD Anderson Cancer Center, Houston, TX USA

## Abstract

Neuronal calcium sensor-1 (NCS-1) has been identified as a binding partner of the taxane, paclitaxel. Our previous study showed that overexpression of NCS-1 increased the efficacy of paclitaxel in vitro, but was associated with poor clinical outcome. Here, we determine if NCS-1 expression is associated with pathological complete response (pCR) to taxane-based neoadjuvant chemotherapy in 105 pre-treatment breast cancer biopsies. Elevated expression of NCS-1 was found to be positively associated with pCR. These results suggest that NCS-1 may be a predictive biomarker for response to taxane-based neoadjuvant chemotherapy in breast cancer.

## Introduction

Neoadjuvant chemotherapy (NAC) is routinely administered in the treatment of breast cancer and response rates are between 15–20%.^1^ Addition of anthracycline-based chemotherapy regimens, which often include taxanes^2^ increases response rates.

Taxanes, a chemotherapeutic agent used to treat breast cancer are effective, but taxanes are associated with toxicities. Moreover, gene expression analyses suggest that some breast cancer subgroups do not benefit from the addition of taxanes to standard anthracycline-based regimens.^3^ A predictive biomarker for taxanes may prevent unnecessary harm by identifying patients susceptible to toxicities.

Taxanes exert anti-proliferative effects by binding to tubulin. Alterations in tubulin represent one proposed mechanism of taxane resistance,^4^ however, additional, alternative mechanisms are needed to understand all the actions of taxanes. Studies have identified several non-tubulin binding partners of taxanes.^5^ We hypothesize that these binding partners represent molecular targets that influence the responses to taxane treatment.

NCS-1, a calcium-binding protein recently identified as a binding partner of paclitaxel,^6^ influences the biological effects of this drug in vitro^7^ and is associated with poor clinical outcome in breast cancer.^8^ Our in vitro studies further demonstrated that overexpression of NCS-1 increased the efficacy of paclitaxel.^8^

Here, we assess NCS-1 expression in a retrospective collection of breast cancer patients treated with neoadjuvant therapy to assess its predictive value.

## Results

**Fig. 1:**
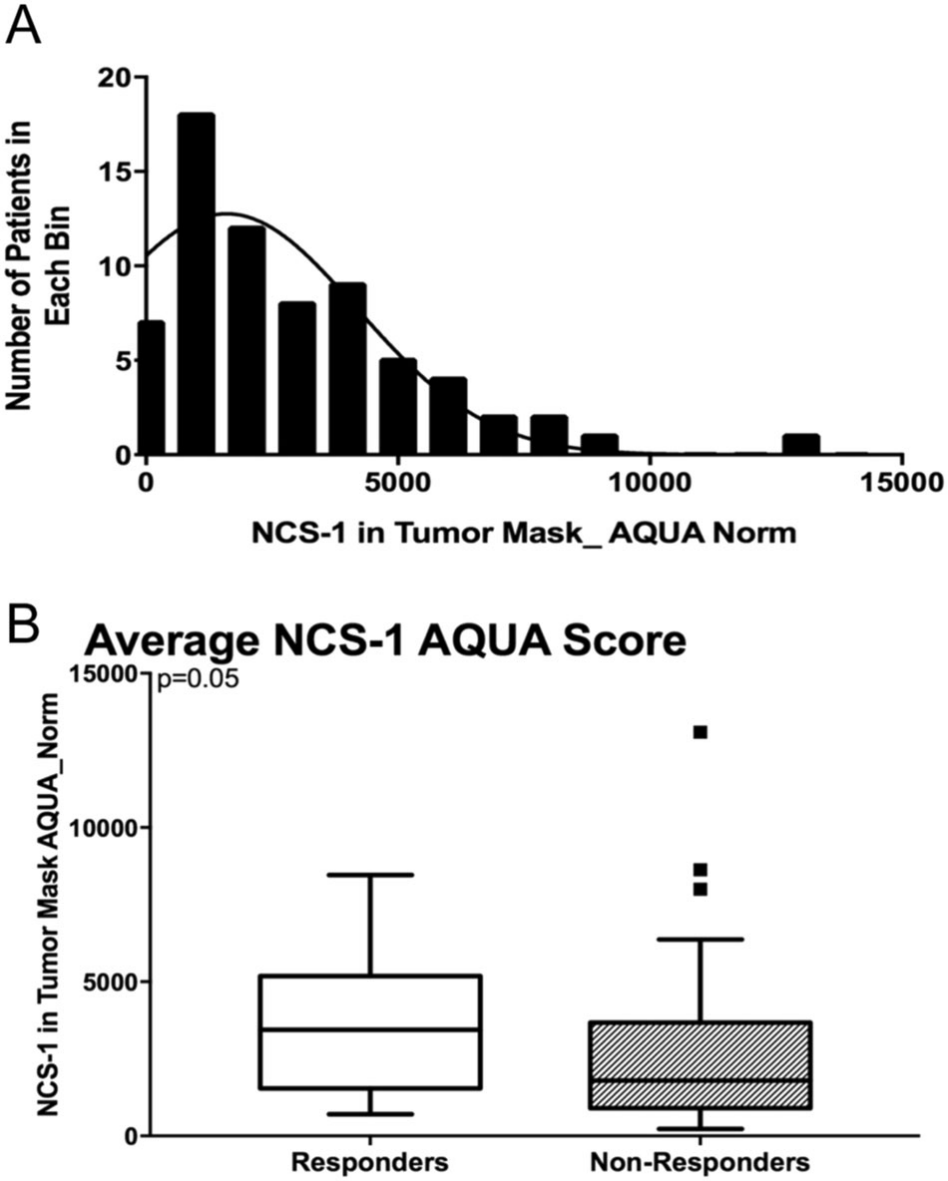
A correlation between NCS-1 expression and pCR. **a** Frequency distribution of average NCS-1 AQUA scores for all fields of view for patients in the neoadjuvant cohort. **b** Box plots of continuous NCS-1 scores in patients that demonstrated pathological complete response (responders) and non-responders

Among this cohort there is a bell-shaped distribution of NCS-1 expression (Fig. 1a) and 26% of patients (18/69) achieved pCR. The mean expression of NCS-1 trended higher among the pCR patients compared to those with residual disease (Fig. 1b).

Based on the analysis of the association between clinicopathological variables and likelihood of pCR (Table 1), the expression of NCS-1 trended towards significance for univariate analysis (OR: 3.17, CI: 0.98–10.19, *p* = 0.06) but was significant by multivariate analysis (OR: 7.75, 95%CI 1.3–76.6, *p* = 0.02).

**Table 1:** Univariate and Multivariate Analysis for likelihood of pCR in neoadjuvant cohort

Characteristics	Univariate		Multivariate	
	OR (95% CI)	*p*-value	OR (95% CI)	*p*-value
Age at diagnosis				
<50	1	0.79	1	0.28
>50	1.217 (0.415–3.571)		2.312 (0.516–11.612)	
Tumor size				
<2 cm	1	0.69	1	0.10
>2 cm	0.681 (0.151–3.070)		0.181 (0.019–1.411)	
Nuclear grade				
Low grade	1	1.00	1	0.13
High grade	0.923 (0.305–2.787)		0.151 (0.007–1.648)	
Nodal status				
Negative	1	**0.002**	1	0.06
Positive	0.136 (0.036–0.511)		0.243 (0.048–1.036)	
ER status				
Negative	1	0.09	1	0.28
Positive	0.333 (0.110–1.009)		0.160 (0.002–3.773)	
PR status				
Negative	1	0.10	1	0.60
Positive	0.378 (0.125–1.140)		0.367 (0.008–15.599)	
HER2 status				
Negative	1	0.69	1	0.97
Positive	1.500 (0.333–6.749)		1.073 (0.026–29.151)	
NCS-1 expression				
Low	1	0.06	1	**0.02**
High	3.165 (0.983–10.194)		7.748 (1.307–76.636)	

*pCR* pathological complete response, *OR* odds ratio, *CI* confidence interval, *ER* estrogen receptor, *PR* progesterone receptor, *HER-2* human epidermal growth factor receptor 2, *NCS-1* neuronal calcium sensor-1

*p* values less than 0.05 are highlighted in bold

A trend between NCS-1 expression and incidence of taxane-induced peripheral neuropathy (TIPN) was previously reported.^8^ Of this cohort 9% reported TIPN (6/69) and 83% of these cases (5/6) belonged to the NCS-1 high-expressing group.

## Discussion

This study shows that NCS-1 is higher in patients that had pCR compared to those with residual disease. Most patients in this cohort (86.6%) received a taxane as part of their treatment strategy. It may seem counterintuitive that NCS-1 appears to facilitate the response of taxanes in vivo because our previous work showed that NCS-1 is a marker of worse prognosis. However, it is likely that NCS-1 is analogous to HER2, where high expression is a marker of poor prognosis, and a marker of therapeutic response to targeted-therapy.

Although this study is underpowered, the observations should be considered hypothesis generating and exploratory. If validated, NCS-1 could be included as a biomarker and may allow for the individualization of patient care and treatment, by identifying patients who are more likely to respond to taxane therapy.

## Methods

### Human subject use and patient cohort

The neoadjuvant cohort consisted of 105 patients who were diagnosed with breast cancer between 2002 and 2010. Details on this cohort were previously published.^9^

### Immunohistochemical staining and quantitative immunofluorescence

Immunohistochemistry was carried as previously described.^8^ Dilutions used for primary antibodies are as follows: mouse anti-cytokeratin (clone AE1/AE3, DAKO; diluted 1:100) and a rabbit anti-NCS-1 (Abcam; diluted 1:1000). The AQUA® method of quantitative immunofluorescence was used for automated image acquisition as previously described.^10^ Only cases with four or more cytokeratin-positive FOVs were included for analysis.

### Statistical analysis

For each patient, AQUA scores for all FOVs were averaged. Statistical analyses were performed using GraphPad Prism and JMP software. Logistic regression was used for multivariate and univariate analysis and statistical significance was determined (*p*-value < 0.05).
